# Assessing Constrained Nuclear–Electronic Orbital Theory for the Floppy **CH**_5_^+^ Ion: Geometry-Dependent Zero-Point Effects on Structure, Hydrogen Rearrangement Dynamics, and Simulated IR Spectra

**DOI:** 10.1021/acs.jpca.6c01211

**Published:** 2026-07-27

**Authors:** Yiwen Wang, Zehua Chen, Yuzhe Zhang, Edwin L. Sibert, Yang Yang

**Affiliations:** Theoretical Chemistry Institute and Department of Chemistry, 5228 University of Wisconsin-Madison, 1101 University Avenue, Madison, Wisconsin 53706, United States

## Abstract

Recently, constrained nuclear–electronic orbital (CNEO) theory has been developed to incorporate nuclear quantum delocalization and zero-point effects into quantum chemistry calculations and *ab initio* molecular simulations. Motivated by discussions with John Stanton, we apply CNEO methods to protonated methane, CH_5_^+^, as a stringent test case for the quantum-corrected effective-potential framework. CH_5_^+^ is a prototypical penta-coordinated nonclassical carbonium ion with a highly anharmonic potential energy surface, many low-lying geometries connected by low barriers, and extensive fluxional motion. Using CNEO, we examine the effective structural features, hydrogen rearrangement dynamics, and simulated IR spectra of CH_5_^+^. The CNEO effective potential energy surface exhibits a minimum with C_2v_ symmetry, which is more symmetric than the minimum-energy eclipsed-C_s_ structure on the conventional potential energy surface. CNEO molecular dynamics predicts more frequent hydrogen rearrangement than conventional *ab initio* molecular dynamics, with pronounced rearrangement observed starting from 50 K. A Fourier-filtered analysis of C–H bond-length distributions along the trajectories, combined with a Gaussian mixture model, reveals three structural and dynamical motifs: equilibrium-like, fluxional, and transition-state hovering configurations. The simulated IR spectra, which are obtained from either harmonic analysis or classical dynamics on the CNEO surface, are compared qualitatively with experimentally observed spectral features. Overall, this work assesses how the computationally efficient CNEO framework captures qualitative structural, dynamical, and spectroscopic trends in the highly fluxional CH_5_^+^, while also highlighting the challenges and shortcomings of this classical-trajectory-based method in a system where fully quantum nuclear effects beyond zero-point effects are important.

## Graphical abstract

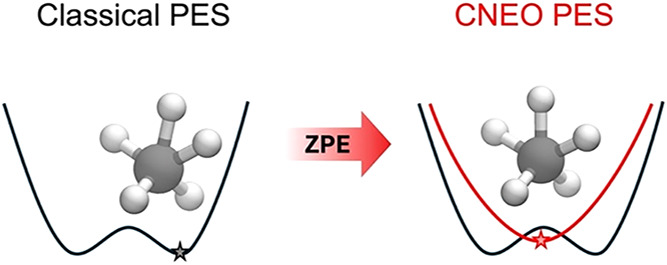

## 1 Introduction

Protonated methane (CH_5_^+^) is a prototypical nonclassical carbonium ion and the parent of penta- and higher-coordinated carbocations.^1,2^ It was first reported in 1952 in mass-spectrometric studies of methane and is recognized as a key intermediate in reactions relevant to astrochemistry and plasma chemistry.^3–5^ Despite its small size, CH_5_^+^ exhibits highly fluxional dynamics and unusually strong coupling between rotational and vibrational motion, making it one of the most challenging small molecular systems for high-resolution spectroscopy and complicating detailed structural, dynamical, and spectroscopic characterization by both theoretical and experimental approaches.^6,7^

From a structural perspective, substantial effort has focused on identifying the equilibrium geometry of this highly fluxional ion. Within the Born–Oppenheimer (BO) framework, electronic structure calculations at various levels of theory have been used to map the potential energy landscape of CH_5_^+^.^8–13^ These studies identified three key low-lying stationary points with similar energies (Figure 1). Among them, the eclipsed-C_s_ shape (Figure 1a) is the only global minimum. It features a three-center-two-electron bonding, with an H_2_ moiety (H2 and H3 in Figure 1a) and a CH_3_ tripod (H1, H4, and H5 in Figure 1a). The other two structures, the staggered-C_s_ structure (Figure 1b) and the C_2v_ structure (Figure 1c), correspond to transition states for hydrogen scrambling, in which hydrogen atoms exchange positions to interconvert between equivalent classical minimum-energy structures. Specifically, the staggered-C_s_ structure is the transition state for pseudorotation of the H_2_ moiety and lies ∼ 0.08 kcal mol^–1^ above the eclipsed-C_s_ minimum, whereas the C_2v_ structure corresponds to the transition state for H-flip motion and lies ∼0.97 kcal mol^–1^ above the eclipsed-C_s_ minimum.^14^

**Figure 1:**
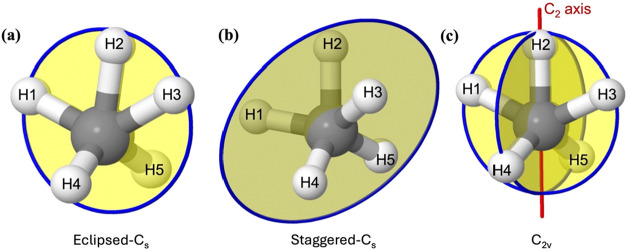
Three low-lying structures of CH_5_^+^ under conventional classical-nuclei treatment, with mirror planes and rotational axis indicated to highlight point-group symmetry properties. (a) Global minimum with eclipsed-*C*_*s*_ symmetry, featuring an H_2_ moiety and CH_3_ tripod. (b) Transition state corresponding to pseudorotation of the H_2_ moiety, with staggered-C_*s*_ symmetry; relative energy ∼0.08 kcal mol^–1^ at the CCSD­(T)/aug-cc-pVTZ level.^14^ (c) Transition state corresponding to H2-flip motion, with C_2v_ symmetry; relative energy ∼0.97 kcal mol^–1^ at the CCSD­(T)/aug-cc-pVTZ level.^14^

However, these structural analyses do not account for nuclear quantum effects (NQEs). For hydrogen rearrangements with such low barriers, NQEs, particularly zero-point effects (ZPEs), can play a significant role. To incorporate NQEs, several quantum simulation approaches have been applied to CH_5_^+^. Early work using path-integral methods found that, despite the pronounced influence of NQEs, the ground-state structure was still dominated by configurations featuring the H_2_ and CH_3_ moieties.^6^ The authors later noted, however, that the simulation time might have been insufficient to achieve complete hydrogen scrambling, and that their conclusions may require re-examination.^15,16^ Subsequent diffusion Monte Carlo (DMC) studies on full-dimensional, permutationally invariant *ab initio* potential energy surfaces provided fully anharmonic zero-point energies, ground-state nuclear wave functions, and bond-length distributions for CH_5_^+^ as well as its isotopologues.^16–18^ These studies showed that the five hydrogen atoms become statistically equivalent in the quantum nuclear distribution, as evidenced by the nearly unimodal C–H bond-length distributions.^16–18^ Similar unimodal C–H bond-length distributions were also observed in later path-integral-based simulations on high-accuracy potential energy surfaces.^19^ In addition, a DMC study yielded three nearly equivalent rotational constants, suggesting that the ground-state structure of CH_5_^+^ is much more symmetric than the eclipsed-C_s_ geometry and highlighting the strong impact of NQEs.^16^

From the perspective of spectroscopic studies, progress has relied on close interplay between experiment and theory. The first high-resolution gas-phase IR spectrum of CH_5_^+^ was reported in 1999,^20^ and subsequent infrared action spectroscopy provided a spectrum of bare CH_5_^+^ over a wider frequency range in 2005.^21^ These measurements stimulated extensive theoretical efforts to interpret the strongly anharmonic vibrational features. DFT-based *ab initio* molecular dynamics (AIMD) simulations at elevated temperatures (∼300 K) were able to reproduce the main experimental signatures even without explicit inclusion of NQEs.^7,21–23^ Later, quantum calculations using DMC together with vibrational self-consistent field/vibrational configuration interaction approaches constructed approximate IR spectra of CH_5_^+^ from vibrational frequencies associated with the three low-lying stationary structures.^24,25^ These spectra showed good agreement with experiment and suggested that contributions from all three structures are needed to reproduce key spectral features. Other quantum-dynamical approaches, including adiabatic centroid molecular dynamics (CMD)^26^ and ring-polymer molecular dynamics (RPMD),^27^ further improved agreement with experiment and provided additional insight into scrambling time scales and isotope effects.

More recently, advances in low-temperature high-resolution spectroscopy have enabled increasingly detailed characterization of the rovibrational features of CH_5_^+^, further revealing its highly fluxional quantum nature and exceptionally strong coupling between rotational and vibrational motion.^28^ Together with the development of increasingly accurate global PESs,^14,19^ these experimental advances have motivated extensive theoretical efforts. Variational rovibrational calculations have provided detailed descriptions of the low-energy rovibrational level structure and permutation-inversion symmetry properties of CH_5_^+^.^30–34^ These studies demonstrated that permutation-inversion symmetry and spin statistics play a central role in determining the physically allowed rovibrational states. In parallel, quantum graph models, constructed from the hydrogen-scrambling pathways and tunneling connectivity between equivalent configurations, have provided simplified but physically insightful descriptions of the low-energy rovibrational structure of CH_5_^+^.^34–37^ Furthermore, molecular superrotor approaches emphasized the importance of simultaneously treating internal and overall rotational motion in CH_5_^+^.^38,39^ Many of these studies also demonstrated the exceptionally strong coupling between rotational and vibrational motion in CH_5_^+^, indicating that explicit consideration of rovibrational coupling is essential for interpreting low-temperature high-resolution rovibrational spectra.

Notably, in these rovibrational calculations, structural interpretations of CH_5_^+^ can depend on the coordinate frame used to analyze the nuclear distribution. For example, in an Eckart body-fixed frame where overall translation and rotation are removed, the apparent nuclear delocalization is limited. This result indicates that CH_5_^+^ can retain recognizable effective structural features in a body-fixed frame, rather than forming a completely diffuse nuclear distribution.^34^

Additionally, isotopic substitution and deuteration have been shown to provide important signatures of nuclear quantum effects in this system. Specifically, existing experimental and theoretical studies indicate that deuteration can affect rovibrational spectral features, site occupation preferences, and hydrogen scrambling time scales.^18,23,25–27^

Recently, our group developed the constrained nuclear-electronic orbital (CNEO) framework, which incorporates NQEs, particularly ZPEs, in quantum chemistry calculations and *ab initio* molecular simulations.^40–42^ It has been applied to study structural, spectroscopic, and dynamical properties of systems containing hydrogen.^40,41,43,44^ The CNEO method belongs to the broad class of multicomponent quantum chemistry theories, which treat both electrons and nuclei quantum mechanically.^45–48^ However, unlike conventional multicomponent approaches, CNEO retains the classical molecular-geometry picture by imposing constraints on the expectation values of the quantum nuclear position operators. The resulting CNEO energy surface is therefore a function of the expectation values of the quantum nuclear position operators and the coordinates of the classical nuclei. Relative to conventional potential energy surfaces, CNEO energy surfaces can be viewed as ZPE-corrected effective potential energy surfaces. On these surfaces, classical molecular dynamics simulations, termed constrained nuclear-electronic orbital molecular dynamics (CNEO-MD),^43,49^ can be performed with minimal additional computational cost.^50^ Our group has demonstrated the capabilities of CNEO methods for vibrational spectra of hydrogen-involving systems,^49–54^ hydrogen-related kinetics and dynamics,^55,56^ and nonadiabatic dynamics.^57^ Furthermore, extensions such as CNEO with implicit solvent model,^58^ CNEO QM/MM,^59^ and periodic CNEO theory^60,61^ have been developed to study hydrogen-related chemistry in complex environments. Notably, CNEO-MD can be viewed as an efficient approximation to CMD, with accuracy that is typically expected to fall between CMD on *ab initio* potential energy surfaces, which has substantially higher cost, and conventional AIMD, which neglects NQEs.^42^

However, CNEO has not previously been applied to CH_5_^+^, in which nuclear quantum effects, hydrogen scrambling, and rovibrational coupling are exceptionally important for a rigorous description. Motivated by discussions with John Stanton, we herein apply CNEO methods to this highly challenging system and assess their performance for this stringent test case. Specifically, we examine how ZPEs and local nuclear delocalization captured by CNEO affect the effective structural features, hydrogen-rearrangement dynamics, and simulated IR spectra of CH_5_^+^. We emphasize that although CNEO incorporates local nuclear quantum delocalization and yields an effective quantum-corrected energy surface, the subsequent nuclear motions are propagated within a classical molecular dynamics framework. Therefore, the present approach is more of a classical method and is not intended to replace the fully quantum treatments of CH_5_^+^. It is also not intended to provide new state-resolved rovibrational assignments. Instead, given the availability of many high-level quantum studies of CH_5_^+^, our goal is to evaluate how effectively the CNEO potential, combined with classical dynamics, can describe relevant structural, dynamical, and spectroscopic trends within the computationally efficient framework. The resulting trajectories also provide an approximate classical picture on hydrogen scrambling dynamics on the CNEO effective surface.

The paper is organized as follows. Section 2 briefly outlines the CNEO theoretical framework and introduces our structural and dynamical classification strategy for CH_5_^+^ configurations. Section 3 describes the computational details. Section 4 presents and discusses the results on structure, dynamics, and spectral features within the CNEO framework. Section 5 provides the conclusions.

## 2 Methods

### 2.1 CNEO Framework

In conventional multicomponent quantum theory,^45–48^ both electrons and a chosen subset of nuclei, such as protons, are treated quantum mechanically within a unified framework, while the remaining nuclei are treated as classical point charges. In such approaches, the resulting energy surface depends only on the coordinates of the classical nuclei, and explicit geometric degrees of freedom associated with the quantum nuclei are therefore not retained. The CNEO framework overcomes this challenge by restoring full-dimensional potential energy surfaces through a constraint imposed on the position expectation value of each quantum nucleus, enforcing it to correspond to classical molecular geometries

$$\left\langle r_{I}\right\rangle =R_{I},\forall I$$ 1

Here, ⟨ ***r**_I_* ⟩ is the position expectation value of the *I*-th quantum nucleus, and ***R***_*I*_ is the corresponding designated classical position. This formulation relies on a distinguishable-particle treatment in which nuclei are described with localized, geometry-centered orbitals, and the orbital position expectation values are constrained to the classical coordinates.

Within CNEO density functional theory (CNEO–DFT), minimizing the total energy with respect to both the nuclear orbitals ({ϕ_*I*_^n^}) and the electronic orbitals (ϕ_*i*_^e^), subject to constraints in eq 1, yields the constrained multicomponent Kohn–Sham equations^40,41^

2 $$\left(-\frac{1}{2}\nabla^{2}+v^{e}\right)\phi_{i}^{e}=\varepsilon_{i}^{e}\phi_{i}^{e}$$

$$\left(-\frac{1}{2m^{n}}\nabla^{2}+v^{n}+f_{I}^{n}\cdot \left(r-R_{I}\right)\right)\phi_{I}^{n}=\varepsilon_{I}^{n}\phi_{I}^{n}$$ 3

Both the electronic and nuclear equations have a kinetic term (∇^2^) and an effective potential term (*v* ^e^ or *v* ^n^). The positional constraint introduces an additional term ***f***_*I*_^n^·(***r*** – ***R***_*I*_) in the nuclear equation, where ***f***_*I*_^n^ is the Lagrange multiplier associated with the constraint on the *I*-th quantum nucleus. Solving these coupled Kohn–Sham equations self-consistently under the constraints yields the multicomponent ground-state orbitals and the corresponding CNEO–DFT total energy. The CNEO energy depends on the constrained expectation values of the quantum nuclear positions as well as on the coordinates of the classical nuclei, thereby giving the ZPE-corrected effective potential energy surface.^40,41^

Under an adiabatic approximation, molecular dynamics on CNEO effective energy surfaces (CNEO-MD) can be justified.^43,62^ The equations of motion for the quantum nuclei are

$$\frac{d}{dt}\left\langle x\right\rangle =\frac{\left\langle p\right\rangle }{m}$$ 4

$$\frac{d}{dt}\left\langle p\right\rangle \approx -\nabla_{\left\langle x\right\rangle }V^{\mathrm{CNEO}}\left(\langle x\rangle \right)$$ 5

where ⟨ ***x*** ⟩ and ⟨ ***p*** ⟩ are the expectation values of the position and momentum operators for the quantum nuclei, and *V* ^CNEO^ is the CNEO–DFT energy surface. For classical nuclei, the equations of motion take the same form, with ⟨ ***x*** ⟩ and ⟨ ***p*** ⟩ in eqs 4 and 5 replaced by the classical ***x*** and ***p***. Consequently, CNEO-MD closely parallels conventional DFT-based AIMD, except that the conventional DFT energy and forces are replaced by the CNEO–DFT energy and forces. This replacement incurs only a small increase in computational cost (<15%), while often providing substantial improvements in stationary structures and vibrational spectra.^50^

### 2.2 Scrambling Event Analysis

Previously, hydrogen rearrangement (scrambling) dynamics has been investigated within a classical picture in which the most stable geometry features an H_2_ moiety formed by H2 and H3 in Figure 1a.^21,22^ The presence of this moiety is commonly taken as the defining feature of a stable CH_5_^+^ configuration and is often identified by locating any pair of hydrogen atoms whose separation is below a threshold (for instance, 1.15 Å).^6^ However, as will be shown later, on the CNEO effective potential energy surface, the most stable geometry of CH_5_^+^ corresponds to a more symmetric global minimum that resembles the C_2v_ stationary point in Figure 1c. This structure lacks the conventional H_2_ moiety. Consequently, the conventional analysis protocol is no longer applicable, and a new classification scheme for CH_5_^+^ configurations is required to analyze the rapid scrambling events.

Instead of characterizing the H_2_ moiety using the H–H distances, we shift our focus to the C–H bond lengths, which, as shown later, can serve as indicators of the local chemical environment for H atoms. However, a main challenge is that raw C–H bond lengths are strongly affected by high-frequency C–H stretching motions. As a result, the vibrational fluctuations can exceed the differences in the average C–H bond lengths associated with distinct environments. These fluctuations make the raw bond lengths unreliable for directly distinguishing H atoms. To address this issue, we remove the high-frequency stretching component by first performing a Fourier transform of each C–H bond-length time series and then applying a low-pass (LP) filter to discard frequencies above 2000 cm^–1^. This LP filtering transforms the highly oscillatory, time-dependent C–H bond lengths (*b*_*i*_(*t*)) into more slowly varying, stretch-free bond lengths $\left({\overset{∼}{b}}_{i}\left(t\right)\right)$

$$\overset{∼}{b}\left(t\right)\equiv \left\{{\overset{∼}{b}}_{i}\left(t\right)\right\}=\left\{\mathrm{LP}\left[b_{i}\left(t\right)\right]\right\}$$ 6

Here, *t* denotes time, *i* labels the H atom indices (*i* = 1,...,5), and ***b* ~**(*t*) is a compact notation for the LP-filtered C–H bond lengths.

Next, using the distribution of all ${\overset{∼}{b}}_{i}\left(t\right)$ values from a set of low-temperature (20 K) simulations, we fit a Gaussian mixture model (GMM) and found that five Gaussian components can approximate the distribution. Two components in the short-bond-length region correspond to H4 and H5 in Figure 1c, which we refer to as the “leg” sites. The remaining three components in the long-bond-length region correspond to H1, H2, and H3, which we refer to as the “head” and “arm” sites. Based on this GMM, for any given filtered C–H bond length ${\tilde{b}}_{i}\left(t\right)$ , the probability that it belongs to each of the five Gaussian components is given by

$$\mathrm{GMM}\left[{\overset{∼}{b}}_{i}\right]=\left(p_{1}\left({\overset{∼}{b}}_{i}\right),p_{2}\left({\overset{∼}{b}}_{i}\right),p_{3}\left({\overset{∼}{b}}_{i}\right),p_{4}\left({\overset{∼}{b}}_{i}\right),p_{5}\left({\overset{∼}{b}}_{i}\right)\right),\overset{5}{\underset{k=1}{\sum }}p_{k}\left({\overset{∼}{b}}_{i}\right)=1$$ 7

where *p*_*k*_(*b̃*_*i*_) is the probability that *b̃*_*i*_ belongs to the *k*-th Gaussian component. For convenience, the components are indexed in order of increasing bond length: *p*_1_ corresponds to the shortest C–H bond-length component, whereas *p*_5_ corresponds to the longest. For each CH_5_^+^ geometry at a given time frame during the dynamics, the five filtered bond lengths each yield five component probabilities, resulting in 25 probabilities in total. These probabilities can be arranged as a 5 × 5 matrix (or, equivalently, a 25-dimensional feature vector) that encodes the structural information on that frame.

As will be shown later, in stable configurations the leg-site distribution is clearly separated from those of the other sites. Therefore, the presence of two and only two leg sites can be used as an indicator of whether the molecule is near its stable configuration. Specifically, because the leg sites have shorter bond lengths, the quantity *q*_*i*_ = *p*_1_(*b̃*_*i*_) + *p*_2_(*b̃*_*i*_) gives the probability that the *i*-th bond is associated with a leg site. When CH_5_^+^ is close to its stable configuration, two H atoms have *q*_*i*_ values close to 1, whereas the remaining three have values close to 0. By choosing a threshold *q*_cutoff_ slightly below 1 (for example, 0.8), the number of bonds associated with leg sites can be computed as *n*_*l*_ = ∑_*i*_Θ­(*q*_*i*_ – *q*_cutoff_), where Θ is the Heaviside function. When CH_5_^+^ is close to stable configurations, *n*_*l*_ = 2, whereas when CH_5_^+^ undergoes a scrambling event, *n*_*l*_ ≠ 2. In this way, *n*_*l*_ provides a convenient indicator for distinguishing stable configurations from fluxional ones during the scrambling process

8 $$n_{l}=\{\begin{array}{ll} 2, & \mathrm{Stable}\\ \mathrm{else}, & \mathrm{Fluxional} \end{array}$$

## 3 Computational Details

We used a locally modified version of PySCF,^63,64^ available through our group’s GitHub repository,^65^ to perform all CNEO–DFT and conventional DFT calculations. Geometry optimizations of both the global minimum and the transition state were carried out using the geomeTRIC optimizer^66^ interfaced with PySCF. All molecular dynamics (MD) simulations were performed using the Atomic Simulation Environment (ASE) package.^67^ Visualization of structures and trajectories was assisted by Jmol^68^ and VMD.^69^

For all calculations, we employed the ωB97MV functional^70^ with the def2-TZVPP electronic basis.^71^ This functional and basis set were chosen based on benchmark harmonic frequency calculations against CCSD­(T) reference results (Table S3). The close agreement with the CCSD­(T) harmonic frequencies suggests that errors from the electronic-structure treatment are relatively small. Additional calculations using larger aug-cc-pVTZ and aug-cc-pVQZ basis sets^72,73^ showed only modest improvements in agreement with CCSD­(T), suggesting that the present computational setup provides a reasonable balance between computational efficiency and electronic-structure accuracy for the present CNEO calculations. This observation is consistent with our group’s previous studies, which have shown that this functional–basis set combination is reliably accurate.^56^ In CNEO–DFT calculations, protons are treated quantum mechanically using the PB4D nuclear basis,^74^ and carbon is treated as a classical point charge. No electron–proton correlation functional is included, as it was found previously that available electron–proton correlation functionals have limited impact on vibrational dynamics.^41^

MD simulations were conducted in the microcanonical ensemble with energy-equipartition presets, denoted as *NVE*-eqp.^51^ In this scheme, multiple simulations are initiated from the optimized global-minimum geometry, with kinetic energy *k*_B_ *T* assigned to each normal mode and velocity directions chosen randomly, where *T* is the system temperature. For each temperature, 50 independent trajectories of 2 ps each were simulated.

To build the GMMs for analyzing hydrogen scrambling events, we collected all C–H bond lengths from CNEO-MD simulations performed at 20 K, where scrambling events were essentially not observed in CNEO simulations. Low-pass Fourier filtering of C–H bond lengths was then performed using the *scipy.signal* package.^75^ Subsequently, GMM fitting was carried out using the *scikit-learn* library.^76^ The resulting GMM model was applied to analyze hydrogen scrambling events across all trajectories.

IR spectra were obtained from the MD trajectories at each simulation temperature by Fourier transforming the dipole derivative autocorrelation function computed from the 50 *NVE* trajectories.

## 4 Results and Discussions

### 4.1 Effective Geometries: Global Minimum and Transition State

Figure 1 shows the three key low-lying stationary geometries of CH_5_^+^ with similar energies on the conventional potential energy surface. As discussed above, the eclipsed-C_s_ structure, which features an H_2_ moiety and a CH_3_ tripod, is the global minimum,^8–13^ whereas the two transition-state structures, the staggered-C_s_ structure and the C_2v_ structure, lie less than 1.0 kcal mol^–1^ above the minimum without considering ZPEs.

Interestingly, on the CNEO–DFT effective potential energy surface, where all five protons are treated quantum mechanically within the distinguishable-nuclei approximation, the most stable structure becomes the more symmetric C_2v_ geometry. Figure 2 shows the optimized structure with the corresponding bond lengths.

**Figure 2:**
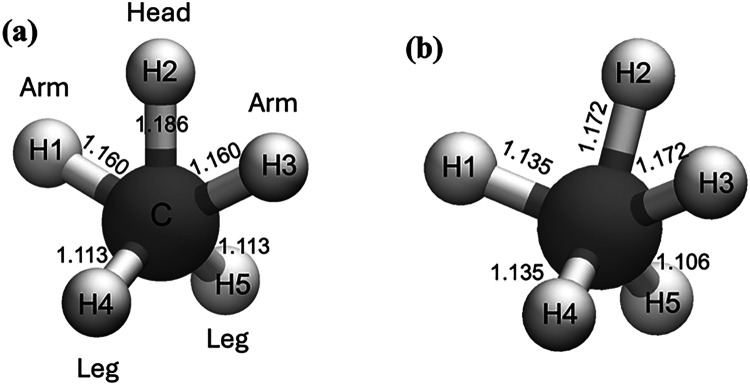
(a) Effective global minimum structure of CH_5_^+^ optimized with CNEO–DFT, exhibiting C_2v_ symmetry. (b) Transition-state structure of CH_5_^+^ with staggered-C_*s*_ symmetry. The relative energy is 0.14 kcal mol^–1^ with respect to the global minimum at the ωB97MV/def2-TZVPP/PB4D level. C–H bond lengths for each structure are indicated (Å).

Notably, several previous studies based on harmonic zero-point energy corrections have suggested that zero-point contributions may shift the relative energies of the three low-lying stationary points, potentially altering their ordering and placing the C_2v_ structure lowest in energy,^11,12,16,18^ although such single-geometry harmonic corrections should be interpreted with caution for the highly fluxional molecule CH_5_^+^.^18^

Additionally, the C_2v_ minimum on the CNEO effective surface may be qualitatively related to previous quantum-graph descriptions of CH_5_^+^.^34,37^ In those studies, C_2v_ configurations were used as vertices, with edges representing internal-rotation pathways that connect these configurations through hydrogen scrambling. Despite the simple treatment, these C_2v_-centered models “surprisingly” reproduced the vibrational energy-level pattern from variational calculations,^34^ suggesting that C_2v_ configurations could provide a useful starting point for the low-energy quantum dynamics of CH_5_^+^. Although quantum-graph model vertices and the CNEO effective surface minimum describe different properties, both point to a notable role of the C_2v_ configuration in CH_5_^+^.

In the optimized C_2v_ geometry by CNEO–DFT, H2 is equidistant from H1 and H3, and the conventional H_2_ and CH_3_ motifs are absent. For clarity, we adopt a simple stick-figure analogy: the C_2v_ structure resembles a person with arms raised at an angle, one leg forward, and the other behind. Guided by this picture, we classify the five hydrogen sites into three types: the head site (H2), the arm sites (H1 and H3), and the leg sites (H4 and H5). Throughout this paper, we use these labels to refer to hydrogen atoms in different local environments. Notably, the two C–H bond lengths associated with the leg sites are significantly shorter than those associated with the head or arm sites.

With the C_2v_ structure as the effective global minimum, H-flip motion becomes a vibrational mode about the minimum and no longer has a distinct transition state. The only remaining transition state is associated with H_2_ pseudorotation. However, unlike the conventional picture involving an H_2_ moiety, the equivalence of H1 and H3 means that pseudorotation can occur either between H2 and H1 or between H2 and H3. The corresponding transition-state structure is shown in Figure 2b, which is the staggered-C_s_ structure and corresponds directly to the staggered-C_s_ structure in Figure 1b. CNEO–DFT calculations show that this staggered-C_s_ structure lies only 0.14 kcal mol^–1^ above the C_2v_ minimum.

Here we emphasize that the C_2v_ minimum discussed here should be interpreted within the scope of the CNEO effective-surface framework. In the present CNEO calculations, the five protons are treated as distinguishable quantum nuclei with localized nuclear orbitals and constrained position expectation values. This representation does not imply that identical protons are physically distinguishable in the exact quantum-mechanical description. Instead, for a fully quantum treatment of CH_5_^+^, the global nuclear wave function must respect complete nuclear permutation-inversion (CNPI) symmetry and the associated nuclear-spin statistical requirements. By contrast, CNEO provides a local, geometry-dependent description of nuclear quantum delocalization and defines an effective potential energy surface for subsequent classical dynamics. Consequently, tunneling splittings, rotational contributions, and nuclear-spin effects in the fully quantum rovibrational spectrum are outside the scope of the CNEO-MD spectral analysis.

Accordingly, the C_2v_ minimum should be understood as a feature of the localized CNEO effective surface, rather than as a direct representation of the fully symmetrized nuclear probability distribution obtained from globally delocalized nuclear wave function methods. It reflects how geometry-dependent quantum delocalization corrections associated with localized nuclear densities modify the effective potential surface within the CNEO description. Because this localized representation focuses on an individual expectation-coordinate geometry rather than the global permutation-inversion space, individual CNEO calculations are characterized by the local point-group symmetry of the constrained geometry, which should be distinguished from the CNPI symmetry classification of globally delocalized nuclear wave functions.^32,33^

To go beyond the zero-temperature optimized-geometry limit and examine finite-temperature effects on the predicted structural features, we further analyzed probability distributions of C–H and H–H distances obtained from CNEO-MD simulations and conventional DFT-based AIMD simulations. These distributions are shown in Figure 3. For CNEO-MD, two types of distributions are reported. The first type, shown in Figure 3a and b, is based on the quantum nuclear position expectation values, whereas the second type, shown in Figure 3c and 3d, is based on the quantum nuclear densities of the underlying CNEO wave function. The expectation-value-based distributions can be obtained directly from the effective geometries along the classical CNEO trajectories. In contrast, the density-based distributions are constructed from the underlying quantum nuclear densities along the trajectories. In analogy with path-integral methods, the first type is more closely related to a centroid distribution, whereas the second type is more closely related to a bead distribution. Because CNEO describes the quantum nuclei through delocalized nuclear densities rather than only through their expectation positions, the density-based distribution provides a more faithful representation of the CNEO nuclear structural distribution, although it is slightly more expensive to obtain.

**Figure 3:**
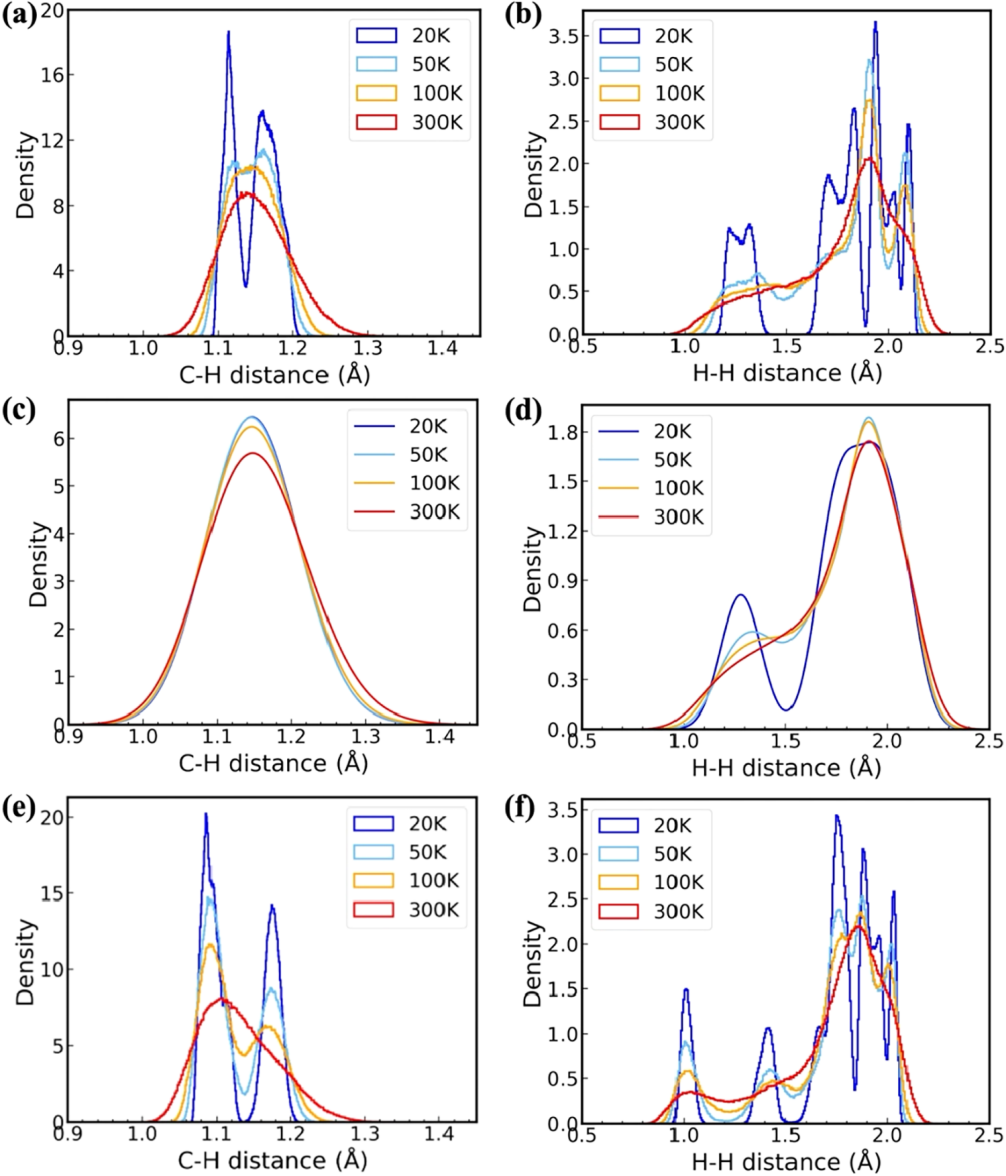
Interatomic distance distributions obtained from CNEO-MD and conventional DFT-based AIMD simulations at different temperatures. The left column shows C–H distance distributions, and the right column shows H–H distance distributions. (a, b) Expectation-value-based CNEO distributions. (c, d) Nuclear-density-based CNEO distributions. (e, f) Conventional AIMD distributions.

In the expectation-value-based CNEO distributions, the C–H and H–H distance distributions retain clear structural features at low simulation temperature, as shown in Figure 3a and 3b. These features are associated with the CNEO C_2v_ effective minimum. As the temperature increases, the peaks broaden and gradually merge, reflecting larger-amplitude hydrogen motion and more extensive exploration of the CNEO effective surface. These expectation-value-based distributions provide a useful picture of the classical dynamics of the nuclear position expectation values, although they do not include the additional spatial delocalization of the quantum nuclear densities contained in the underlying CNEO wave function.

When the underlying nuclear densities are taken into consideration, the C–H distance distribution changes substantially. As shown in Figure 3c, the density-based CNEO C–H distribution is broad and unimodal already at 20 K, indicating that the distinct local C–H environments are largely washed out in the quantum nuclear distribution. As the temperature increases, only modest additional broadening is observed. This unimodal distribution resembles the C–H distributions reported in DMC and path-integral simulations.^16,17,19^ For H–H distances, the density-based CNEO distribution also shows substantial broadening relative to the expectation-value-based distribution. At 20 K, the CNEO H–H distribution in Figure 3d is already much broader than the corresponding distribution in Figure 3b and exhibits a broad two-feature pattern. As the temperature increases, the separation between the two features becomes less pronounced, giving better qualitative agreement with the DMC and path-integral distributions.^16,17,19^ These good results from the underlying CNEO quantum nuclear distributions are consistent with one of our group’s previous studies, in which real-time CNEO proton distributions were shown to accurately reproduce quantum reference distributions in nonadiabatic dynamics for a model system.^57^

In contrast, conventional DFT-based AIMD retains distinct geometry-specific C–H peaks at low and intermediate temperatures and approaches a similarly broad distribution only at much higher simulation temperature, as shown in Figure 3e. A similar trend is observed for the H–H distance distribution, as shown in Figure 3f, where a comparable broad two-feature pattern appears only near 300 K in conventional AIMD. This improved performance at elevated temperature is consistent with previous studies showing that classical MD on an accurate PES, when performed at approximately the quantum zero-point energy, can reproduce important features of the total C–H and H–H structural distributions of CH_5_^+^, although isotope-resolved distributions for deuterated isotopologues reveal clear differences between the classical and quantum descriptions.^17^ Notably, even when comparing only the expectation-value-based CNEO distributions with the conventional AIMD distributions, CNEO gives more delocalized structural distributions. This behavior is consistent with the reduced effective barriers on the CNEO surface,^55,56^ which arise from the geometry-dependent zero-point effects and nuclear delocalization effects included through the underlying quantum nuclear description.

However, we note that the agreement between CNEO and previous DMC and path-integral results is not as close for H–H distances as it is for C–H distances. In the DMC and path-integral reference distributions, the short-distance H–H feature appears as a weak shoulder near 1.0 Å that is smoothly connected to the dominant longer-distance peak.^16,17,19^ In the 20 K density-based CNEO distribution, the two features remain more clearly separated, and the short-distance feature is centered at somewhat longer H–H distances, with relatively low probability near 1.0 Å. These differences likely arise from the local nature of the CNEO nuclear wave function and the classical nature of the MD simulation. Specifically, the density-based distribution includes only limited local spatial delocalization of each quantum proton, while still being constructed along classical trajectories of the nuclear position expectation values, which mainly oscillate around the CNEO C_2v_ effective minimum and do not fully explore the broader hydrogen-rearrangement configurational space at low temperature.

### 4.2 Dynamics

Beyond the stationary effective structure, hydrogen scrambling is a key dynamical feature of CH_5_^+^ and requires a detailed understanding. Previous studies have identified two main scrambling pathways:^12,22^ H-flip via the C_2v_ transition state, and H_2_ pseudorotation via the staggered-C_s_ transition state. Because both pathways involve breaking and rearranging the H_2_ moiety, prior work typically defines a stable CH_5_^+^ configuration by the presence of an H_2_ moiety, which was identified when a pair of hydrogen atoms had an H–H separation shorter than a threshold (for instance 1.15 Å).^6^ Using this criterion, scrambling frequencies, time scales, and isotope effects have been investigated within the conventional electronic-structure framework.^6,7,21–23,26,77^

However, because CNEO–DFT predicts a different global minimum effective structure that lacks the H_2_ moiety, the conventional H–H distance criterion is no longer applicable. As a result, both the definition of a scrambling event and the analysis of scrambling pathways must be revisited within the CNEO framework. Motivated by our observation that the two C–H bonds associated with the leg sites are significantly shorter than those associated with the head and arm sites in C_2v_ configurations, we instead characterize CH_5_^+^ conformations using the presence of two leg sites as the indicator, as detailed in Section 2.2.

#### 4.2.1 Low-Temperature Dynamics for GMM Construction

We first investigate the low-temperature classical dynamics on the CNEO-PES, where available classical kinetic energy is generally insufficient to cross the hydrogen-scrambling barrier. Specifically, no scrambling events were observed in the finite 2 ps CNEO-MD trajectories initialized with kinetic energies corresponding to *k*_B_ *T* at *T* = 20 K. We note that this result should not be interpreted as evidence that fluxional scrambling dynamics are absent in the real CH_5_^+^ system at 20 K. Rather, it reflects the limitations of all classical dynamical treatments and the finite trajectory length used here. Within classical dynamics, scrambling, if it were to occur, would require a rare barrier-crossing event whereas in a fully quantum-mechanical description the system may access equivalent configurations through tunneling.

Figure 4a shows the five time-dependent C–H bond lengths from an example 20 K trajectory. As a reference, the three black dashed lines (from longest to shortest) indicate the C–H bond lengths of the head, arm, and leg sites, respectively, in the C_2v_ geometry. During the dynamics, the bond lengths oscillate significantly about their equilibrium values due to C–H stretching motions. Although the separation between the arm and leg sites is mostly clear, the instantaneous bond lengths associated with the head and arm sites can exhibit substantial overlap even though scrambling is not observed. These high-frequency oscillations therefore make direct classification based on instantaneous C–H bond lengths noisy and unreliable. To suppress this noise, we apply a low-pass filter to the time-dependent bond lengths and remove frequency components above 2000 cm^–1^. This procedure retains only the low-frequency vibrations and can more accurately distinguish the bonding environments. As shown in Figure 4b, the filtered bond lengths display much clearer separation among the head, arm, and leg regions, with especially distinct separation of the leg sites from the others.

**Figure 4:**
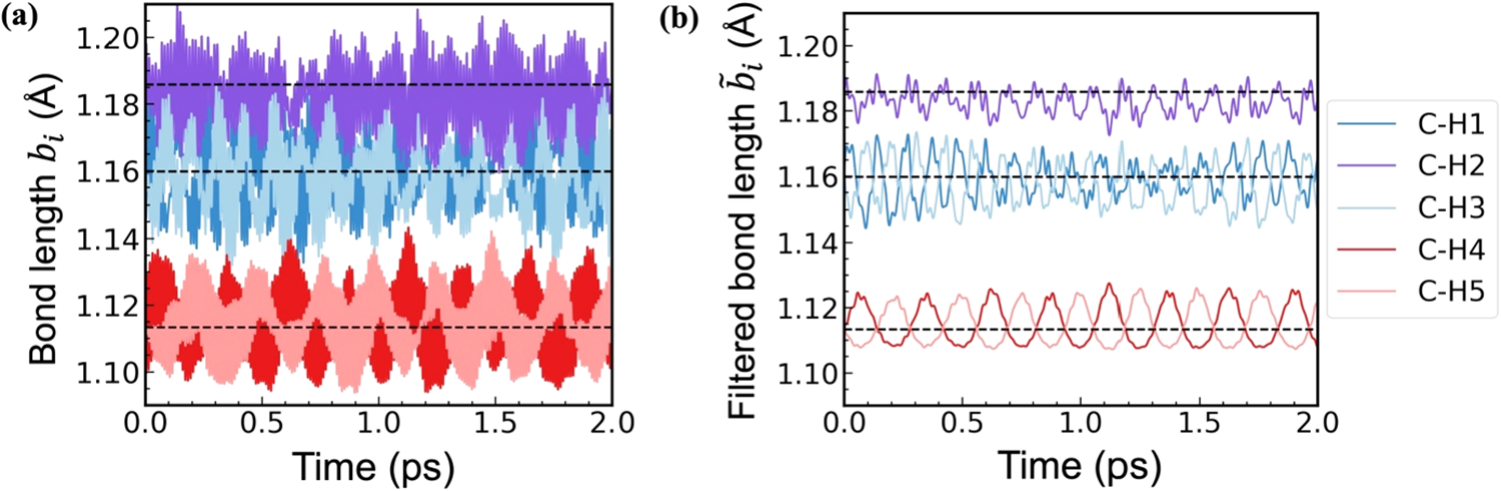
Five instantaneous (a) C–H bond lengths and (b) filtered C–H bond lengths along one representative 2 ps simulation trajectory at 20 K under the *NVE*-eqp scheme. The hydrogen indices correspond to the labeling in Figure 2. Three distinct regions are clearly observed in both cases, representing the head, arm, and leg bonding sites. Black dashed lines (from longest to shortest) indicate the C–H bond lengths of the head, arm, and leg sites, respectively, in the global-minimum geometry.

Because scrambling events are not observed at 20 K, these trajectories provide a reference ensemble of fluctuations around the C_2v_ structure. We therefore use them to construct a GMM for characterizing stable configurations. In principle, the unfiltered bond lengths may also be used (see the Section S4 for details); however, because the filtered bond lengths provide clearer separation among environments, we use the filtered data here. The distribution of filtered C–H bond lengths from these trajectories is shown as the gray shaded region in Figure 5. The distribution resolves into three main regions corresponding to the head, arm, and leg sites in the stick-figure model. In addition, both the arm and leg regions exhibit two peaks. This shape arises because the residual low-frequency motions modulate the bond lengths (Figure 4b), and the bond lengths spend more time near the turning points of the oscillation. This effect is less pronounced for the head site, for which the C–H bond-length distribution is largely unimodal. Although these peak shapes are not strictly Gaussian, we fit the total distribution with a five-component GMM for simplicity. The resulting fit is shown in Figure 5, with the individual components plotted as colored curves.

**Figure 5:**
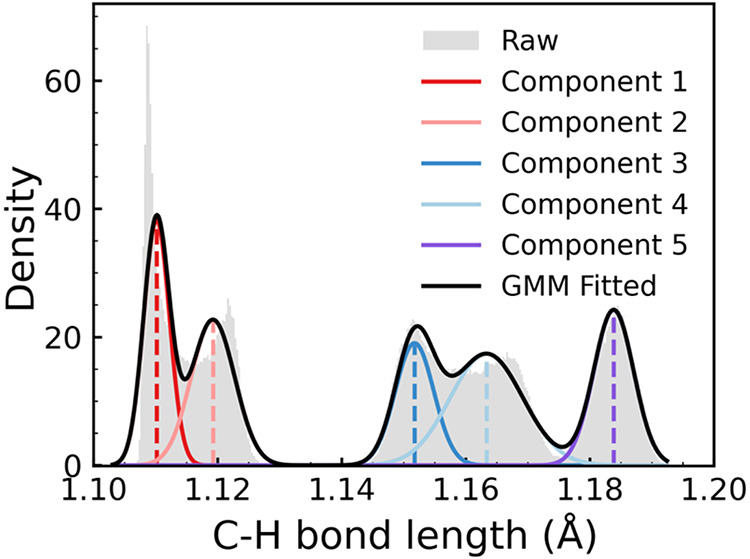
GMM fitting of the filtered C–H bond length distribution. The shaded area represents the collected filtered C–H bond length data from 50 trajectories at 20 K, each containing 4000 configurations, yielding a total of 200,000 bond lengths. Individual Gaussian components from the GMM fit are shown as colored curves, and their summed profile is represented by the solid black line, corresponding to the overall fitted distribution. Three distinct regions are identified: two shorter components correspond to the leg sites, two intermediate components represent the arm sites, and the longest-bond component corresponds to the head site.

#### 4.2.2 Higher-Temperature Dynamics with Frequent Scrambling

We next examine the dynamics at elevated temperature by applying the fitted GMM to the C–H bond lengths for trajectories at 50 K, where hydrogen scrambling events become frequently observable within the finite 2 ps CNEO-MD trajectories. Figure 6a shows the filtered C–H bond lengths from a representative 50 K trajectory. At this temperature, the system has sufficient energy to access scrambling events, while the scrambling rate remains low enough to allow clear visualization and detailed comparison between the descriptors and the underlying structural evolution.

**Figure 6:**
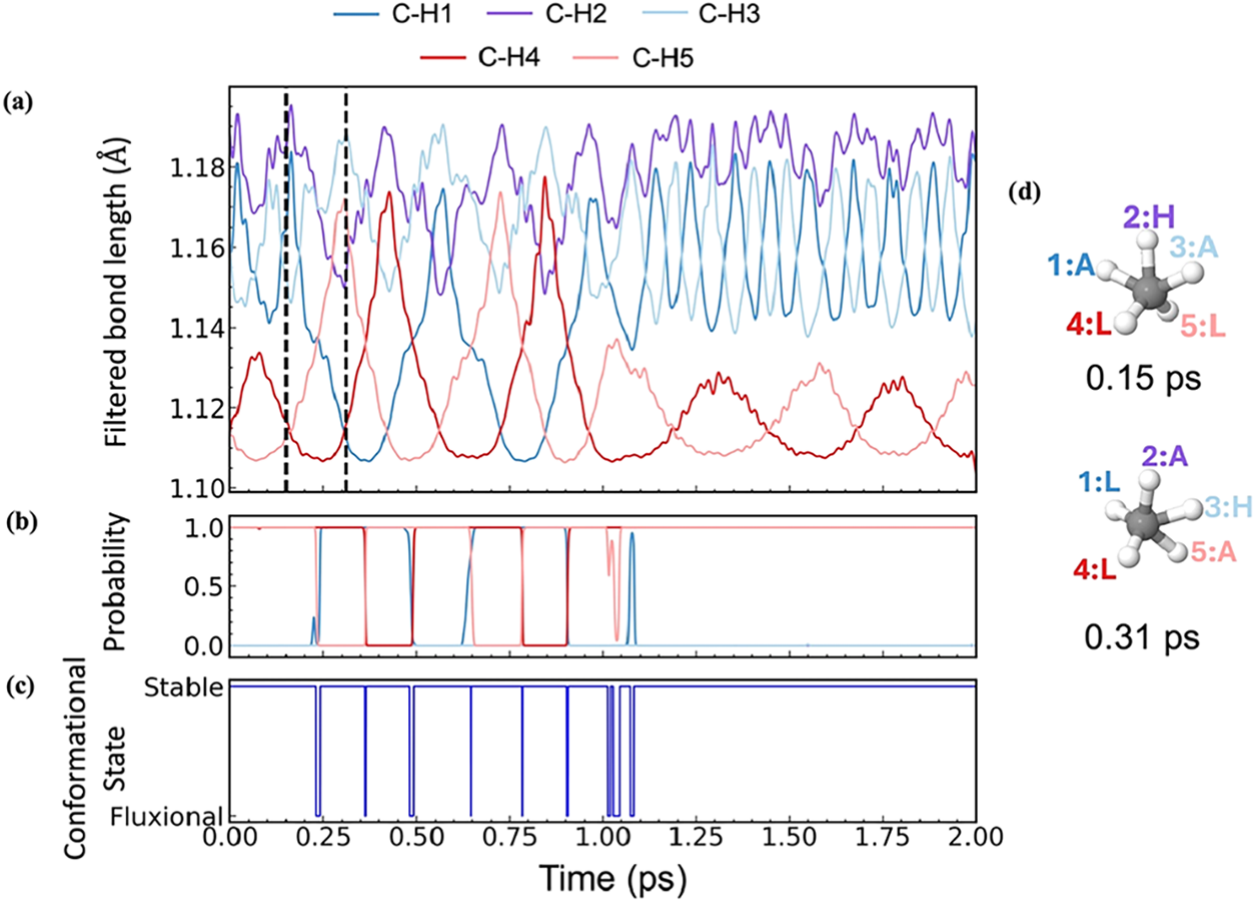
A representative 50 K trajectory and its GMM-based analysis. (a) Filtered C–H bond lengths for all five hydrogens. Black dashed lines indicate the times at which the snapshots shown in panel (d) are taken. (b) GMM-predicted probabilities that each bond corresponds to either of the two leg sites. (c) Conformational state assignments obtained by counting the number of bonds whose leg-site probability exceeds the chosen cutoff. Hydrogen indices follow the labeling scheme in Figure 2. (d) Two representative structural snapshots taken before and after a scrambling event, showing the rearrangement of hydrogen bonding environments (H: head site; L: leg site; A: arm site).

Two major dynamical patterns can be observed in this representative 2 ps trajectory. First, as exemplified by the segment from 1.1 to 2.0 ps, the behavior resembles that at 20 K: the head, arm, and leg sites oscillate within three relatively well-separated regions, with particularly clear separation of the leg sites from the others. This behavior indicates that CH_5_^+^ remains near the effective C_2v_ structure during this time window, without scrambling.

The second pattern is characterized by crossings between the leg and arm regions, as well as between the head and arm regions, which occur several times between 0.2 and 1.0 ps. By inspecting the structural changes before and after the crossings (Figure S3), we find that each set of crossings corresponds to a hydrogen-scrambling event that proceeds through a transition-state geometry resembling the staggered-C_s_ structure. Specifically, as illustrated by the representative structures before and after a scrambling event (dashed lines denote the times and structures indicated in Figure 6d), the original head site becomes an arm site; the two original arm sites become a leg site and the new head site, respectively; and one of the original leg sites becomes an arm site.

These scrambling events are also captured by the GMM and the *n*_*l*_ indicator described in Sec. 2.2. Figure 6b shows the GMM-predicted probability that each bond belongs to a leg site, and Figure 6c shows the resulting configuration classification based on *n*_*l*_. Each time an arm-leg-bond-length crossing occurs in Figure 6a, corresponding changes in leg-site assignment are observed in Figure 6b, and a scrambling event is identified in Figure 6c. The close agreement among these indicators demonstrates that the obtained GMM can reliably distinguish stable and fluxional configurations as well as capture scrambling events.

We note one additional feature in this representative trajectory. Between the scrambling-rich region (0.2–1.0 ps) and the stable region (1.1–2.0 ps), there is a short interval (1.0–1.1 ps) that corresponds to attempted but incomplete scrambling events. In this interval, the GMM-predicted leg probabilities do not fully reach values near 1 or 0, and it corresponds to a transient relaxation region following highly active dynamics. The presence and duration of such a relaxation interval vary among trajectories and can be longer, shorter, or absent.

#### 4.2.3 Special Dynamics: Transition-State Hovering

In addition to the two dynamical patterns discussed above, some 50 K trajectories reveal a less common but distinct behavior that we term transition-state hovering. This dynamics is characterized by the prolonged persistence of a single short C–H bond after LP filtering, while the remaining four longer C–H bonds actively fluctuate. An example is shown in the 0.2–0.9 ps time window of Figure 7a with an animation of the nuclear motion provided in the Supporting Information. During this period, the CH_5_^+^ structure remains closely aligned with the transition-state geometry shown in Figure 2b, with the C–H bond in the mirror plane remaining as the single short leg while the other four C–H bonds continuously exchange their relative lengths. As a result, the structure hovers among equivalent transition-state geometries before eventually trapped at a low-energy C_2v_ structure. In the representative trajectory, the hydrogen atom located in the molecular mirror plane (e.g., H5 in Figure 2 and H2 in this trajectory segment) remains the single leg site, whereas the four out-of-plane hydrogens interchange their roles with large bond-length fluctuations.

**Figure 7:**
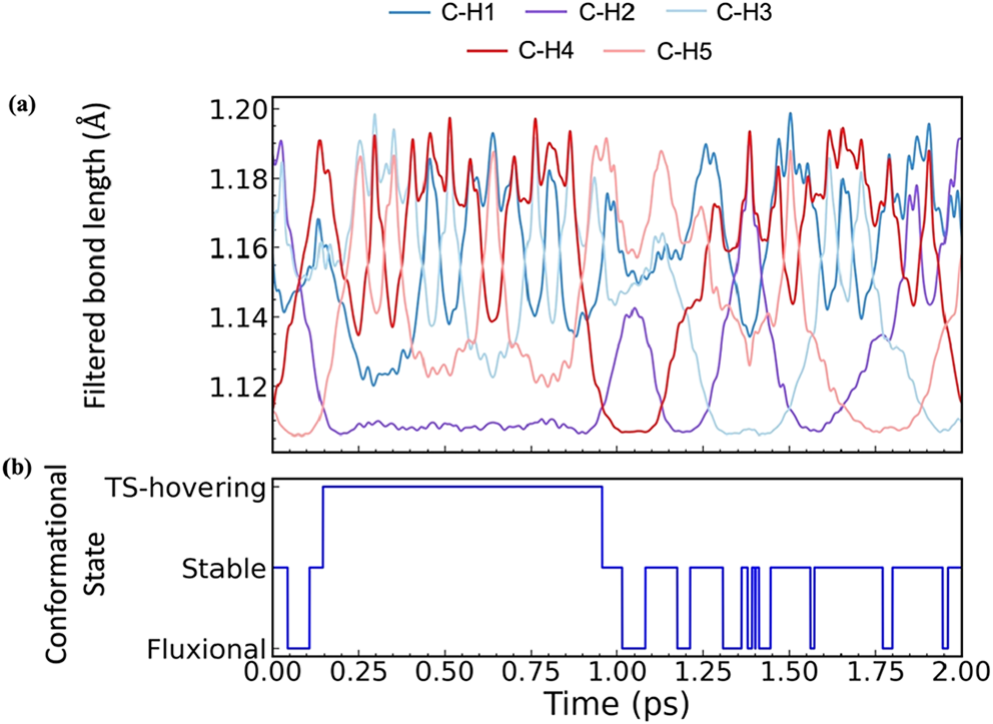
(a) Filtered C–H bond lengths along a representative 50 K trajectory illustrating transition-state-hovering dynamics. The 0.2–0.9 ps time window, during which one C–H bond remains distinctly shorter than the other four, corresponds to CH_5_^+^ hovering among classically equivalent transition-state geometries. (b) Conformation assignments for the same trajectory.

When analyzed using the GMM-based classification scheme, these transition-state-hovering structures can be misidentified as stable configurations, particularly when one of the four fluctuating bonds transiently shortens toward the leg-site region (Figure S4), which leads the model to incorrectly assign two leg sites. In this situation, a scheme based solely on instantaneous structural features cannot reliably detect the hovering pattern. To address this limitation, we exploit the persistence of the single-leg bond and introduce an additional temporal criterion: a configuration is classified as transition-state hovering when the index of the shortest filtered C–H bond remains unchanged for a specified duration (for example, 0.3 ps). Applying this criterion allows the long-lived hovering pattern to be distinguished from genuine stable configurations, and the final assignments for the representative trajectory are shown in Figure 7b. Across the 50 K trajectories analyzed in this study, transition-state hovering accounts for approximately 20% of the total simulation time.

### 4.3 IR Spectra

After characterizing the new stable geometry and the associated scrambling dynamics, we next examine the IR spectra within the CNEO framework. We note that the spectra discussed below are obtained on the CNEO effective potential surface, either from local harmonic analysis based on the C_2v_ structure or from Fourier transforms of dipole derivative autocorrelation functions along classical molecular dynamics trajectories. They are not intended to provide rigorous assignments of experimental features to exact quantum rovibrational eigenstate transitions. Instead, they represent approximate IR spectra generated within the CNEO framework and are used for qualitative comparison with the experimental results. This distinction is particularly important for CH_5_^+^, where strong hydrogen scrambling and rovibrational coupling make conventional geometry-specific assignments questionable.

The CNEO harmonic spectrum based on the C_2v_ structure (Figure 8e; frequencies listed in Table S2 and vibrational modes visualized in Figure S1) shows two major peaks in the midfrequency region and five peaks in the high-frequency region. In the midfrequency region, the peaks at 1056 and 1230 cm^–1^ correspond to (i) symmetric bending dominated by the two arm sites and (ii) a rocking motion dominated by the leg sites, respectively. In the high-frequency region, the five peaks correspond to the out-of-phase symmetric stretch of the head and arm sites (2503 cm^–1^), the in-phase symmetric stretch of the head and arm sites (2609 cm^–1^), the asymmetric stretch of the arm sites (2769 cm^–1^), the symmetric stretch of the leg sites (2966 cm^–1^), and the asymmetric stretch of the leg sites (3065 cm^–1^). Among these modes, the asymmetric stretch of the arm sites carries the largest intensity.

**Figure 8:**
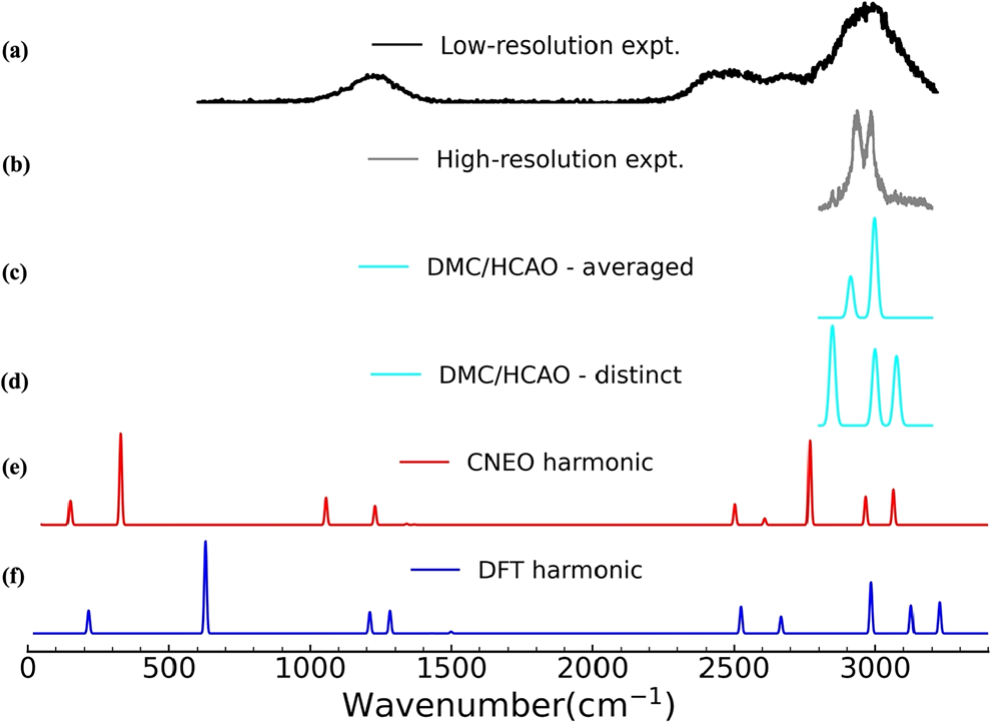
Comparison of experimental and calculated IR spectra of CH_5_^+^. (a) Lower-resolution experimental infrared action spectrum at 110 K, digitized from ref 21. (b) High-resolution IR spectrum of the high-frequency region measured in helium droplets at 0.38 K, digitized from ref 79. (c) Calculated IR spectrum reported by Moonkaen and McCoy, based on DMC simulations combined with harmonically coupled anharmonic oscillator (HCAO) model, in which the CH bonds within the H_2_ moiety and the CH_3_ group were made equivalent separately. Data digitized from ref 78. (d) Same as (c), but all hydrogen atoms are treated as distinct. (e) Calculated spectrum from CNEO–DFT harmonic analysis. (f) Calculated spectrum from DFT harmonic analysis.

Overall, the character of these modes closely parallels the assignments from conventional DFT harmonic analysis, even though the underlying minimum-energy structures slightly differ. In fact, the conventional DFT minimum can be viewed as a “tilted-head” variant of the stick-figure picture, where the head site tilts toward one arm site to form the H_2_ moiety. Within this perspective, the vibrational modes map naturally onto those of the “straight-head” C_2v_ picture obtained with CNEO. A notable difference is that CNEO predicts a significant redshift of the vibrational frequencies, particularly for stretching modes. The largest shift occurs for the asymmetric stretch of the arm sites: in conventional DFT harmonic analysis this mode appears above 3000 cm^–1^ (Figure 8e), whereas in CNEO it shifts downward by approximately 300 cm^–1^. This shift is so large that it does not align well with the most intense experimental peak. However, the three highest-frequency peaks from the CNEO harmonic spectrum agree well in position with the DMC-based high-resolution spectrum reported by Moonkaen and McCoy when all hydrogen atoms are treated as distinct (Figure 8d).^78^ This agreement suggests that the harmonic frequencies of the CNEO C_2v_ structure capture several underlying vibrational features of the system. However, reproducing the double-peak structure observed in the helium nanodroplet experiment^79^ and in the symmetry-averaged DMC/HCAO spectrum (Figure 8c)^78^ likely requires a more complete treatment of the globally delocalized nuclear distribution and symmetry averaging. These effects are not captured by the present localized CNEO harmonic analysis and are more appropriately described using higher-level quantum nuclear-motion methods.^31–33,78^

Beyond the mid- and high-frequency regions discussed above, one additional mode is worth discussing: a head-site proton-transfer motion. In the CNEO “straight-head” picture, this mode corresponds to the head hydrogen swinging back and forth between two positions closer to the two arm sites, that is, a proton-transfer-like motion along the head–arm direction. CNEO harmonic analysis with the ωB97MV/def2-TZVPP electronic functional predicts a frequency of 330 cm^–1^ for this mode. In contrast, in the conventional electronic-structure picture with a tilted head site, this proton-transfer mode is absent, and the head hydrogen undergoes only a more localized vibrational motion. The frequency of this local mode is sensitive to the electronic-structure method, yielding 815 cm^–1^ at CCSD­(T)/def2-TZVPP and 628 cm^–1^ at ωB97MV/def2-TZVPP (Table S3).

This qualitative difference closely resembles the shared-proton H_3_O_2_^–^ case, where CNEO predicts an equally shared proton with a vibrational frequency close to experiment, whereas conventional DFT favors a more localized proton and substantially overestimates the corresponding frequency.^50^ For CH_5_^+^, the reported experimental spectrum extends down to ∼500 cm^–1^ and shows no clear feature in the 500–1000 cm^–1^ region. In addition, previous CMD and RPMD simulations that excellently reproduce the measured spectrum show little intensity until frequencies approach the lower end of the experimental window, with the relevant feature anticipated to peak below 500 cm^–1^.^26,27^ Together, these observations are more consistent with the lower-frequency CNEO prediction.

However, at the same time, we note an experimental caveat: as noted by the authors in the experimental study, the signal sensitivity in the photo detachment action spectroscopy decreases substantially below ∼1000 cm^–1^,^21^ so a weak feature in the 500–1000 cm^–1^ region cannot be ruled out. A more definitive distinction between the straight-head CNEO picture and the tilted-head conventional picture would therefore benefit from measurements with higher sensitivity in the 300–1000 cm^–1^ range.

In our experience, MD-based spectra often provide improved agreement and additional insight beyond harmonic analysis because they incorporate anharmonicity and intermode coupling effects.^49–51,53^ We reuse the same *NVE*-eqp trajectories employed for the dynamics analysis to compute the IR spectra. Figure 9 compares the CNEO-MD simulated IR spectra at several simulation temperatures with the experimental spectrum, and Figure 10 shows the corresponding comparison for conventional AIMD.

**Figure 9:**
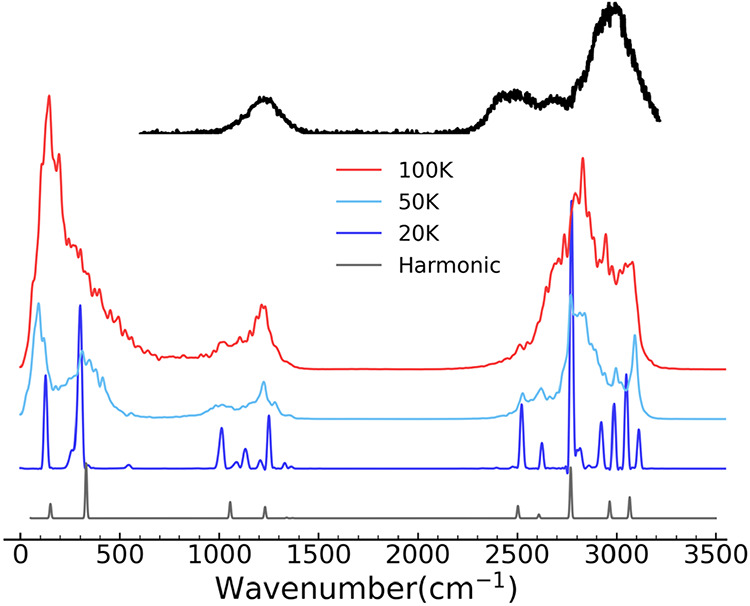
IR spectra of CH_5_^+^ from CNEO–DFT harmonic analysis and CNEO-MD simulations at 20, 50, and 100 K, compared with the experimental IR spectrum digitized from ref 21. (black solid line).

**Figure 10:**
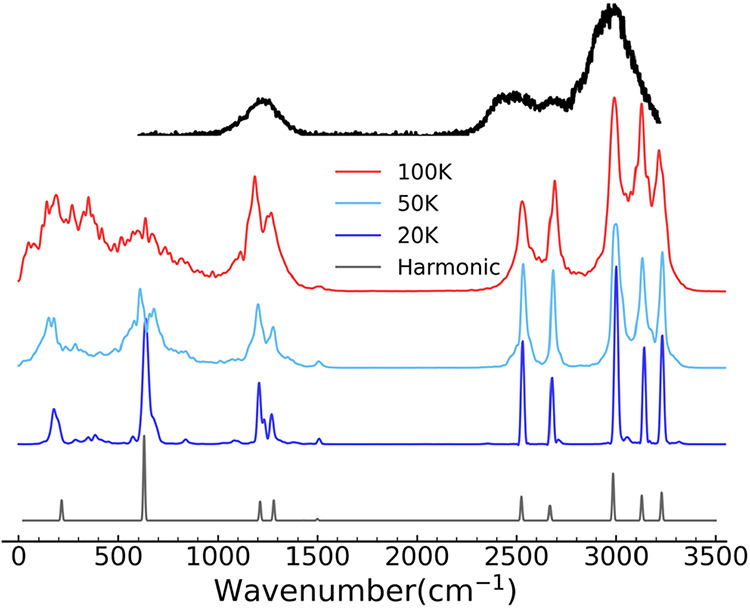
IR spectra of CH_5_^+^ from DFT harmonic analysis and AIMD simulations at 20, 50, and 100 K, compared with the experimental IR spectrum digitized from ref 21. (black solid line).

At 20 K, the simulated spectrum (Figure 9, blue) exhibits distinct, well-resolved peaks and remains broadly consistent with the harmonic picture. A key difference emerges in the high-frequency region, where the simulated spectrum displays seven discernible peaks rather than the five stretching modes predicted by harmonic analysis. Two additional features appear near 2920 and 3120 cm^–1^. These frequencies are close to sums of a stretching mode and a low-frequency mode, such as 2769 + 151 cm^–1^ and 2966 + 151 cm^–1^, suggesting that they may arise from effective classical coupling between C–H stretching motion and low-frequency hindered rotational motion. However, for this highly fluxional molecule, such assignments of classical spectral features should be interpreted with caution, and more accurate quantum methods would be needed for definitive interpretation.

When the simulation temperature increases to 50 K (Figure 9, light blue), stronger intermode coupling effects and more frequent hydrogen scrambling events lead to substantial spectral broadening and reduced peak resolution. In the midfrequency region, the sharp 1056 cm^–1^ feature diminishes into a weak shoulder, whereas the 1230 cm^–1^ feature becomes dominant. In the high-frequency region, the distinct peaks observed at 20 K merge into a broad band. The 2503 and 2609 cm^–1^ features remain visible within the broad envelope, and the dominant intensity is centered near the 2769 cm^–1^ feature. The highest-frequency part of the spectrum retains a feature around 3100 cm^–1^, which is associated with leg-site stretching. Overall, the 50 K spectrum exhibits broader and less structured features that qualitatively resemble the lower-resolution experimental spectrum.

At the same time, the increased frequency of scrambling events introduces additional limitations for the present dipole-autocorrelation-based analysis. The quantum–classical correspondence underlying this approach is best justified for vibrational motions around a geometry local minimum, but the frequent escape from the local minimum weakens this justification and makes detailed interpretation of individual spectral features using classical dynamics less reliable.

We also examined the spectra at 100 K (Figure 9, red). At this higher temperature, the CNEO spectrum becomes even broader. In particular, the high-frequency region largely collapses into a single broad band. The features associated with the in-phase and out-of-phase symmetric stretches of the head and arm sites become difficult to observe because the rapid hydrogen rearrangement blurs the distinction among local hydrogen environments in the classical dynamics on CNEO effective surface. In contrast, conventional DFT-based AIMD exhibits comparatively infrequent scrambling over similar simulation time scales, allowing more localized spectral features to remain visible (Figure 10, red). In fact, conventional AIMD has been reported to reproduce experimental spectra with good agreement at 300 K,^7,21,22^ despite the physical expectation that substantial scrambling should occur. These observations suggest that the absence of zero-point effects in conventional AIMD may keep the dynamics close to the local-minimum regime and thereby significantly underestimate the scrambling frequency.

Another possible source of the discrepancy in the CNEO spectra is the nature of the CNEO effective energy surface itself. Because the CNEO surface is constructed under an adiabatic approximation, it can be viewed as a low-temperature effective surface. In principle, as the temperature increases, the effective surface should evolve and approach the classical potential energy surface.^42^ This temperature dependence is captured naturally in CMD, whereas in the current formulation the CNEO effective surface is temperature independent. Because 100 K can be considered a high temperature for hydrogen scrambling—unlike many hydrogen-related vibrational problems where even room temperature remains effectively low—the error introduced by using a fixed low-temperature effective surface may become more significant. This limitation, as a possible source of discrepancy, motivates future development of temperature-dependent CNEO effective surfaces.^42^

## 5 Conclusions

In this work, we applied the CNEO framework to the prototypical fluxional carbonium ion CH_5_^+^ and investigated the effective structure, hydrogen-rearrangement dynamics, and simulated vibrational spectra. Because extensive experimental data and high-level computational results are available from variational quantum wave function methods, DMC calculations, and path-integral-based approaches, CH_5_^+^ provides a stringent test case for assessing the strengths and limitations of CNEO. Our results examine how this computationally efficient classical dynamics framework with local nuclear quantum delocalization corrections captures qualitative structural, dynamical, and spectroscopic trends in a system where fully quantum nuclear-motion effects are exceptionally important.

For the stationary effective structure, CNEO–DFT predicts a minimum effective structure with C_2v_ symmetry, in contrast to the conventional DFT minimum at the eclipsed-C_s_ geometry. In this picture, the traditional H_2_–CH_3_ motif is absent, and the system is more naturally described using a stick-figure picture with head, arm, and leg sites. The staggered-C_s_ structure associated with pseudorotation becomes the only transition state and lies only 0.14 kcal mol^–1^ above the C_2v_ minimum. Finite-temperature CNEO-MD simulations further show that local proton delocalization substantially broadens the C–H and H–H structural distributions relative to conventional AIMD. This effect is especially clear in the nuclear-density-based C–H distribution, which becomes nearly unimodal already at low simulation temperature, in qualitative agreement with previous DMC and path-integral results. The H–H distribution also shows substantial broadening relative to AIMD and develops the broad two-feature pattern characteristic of quantum reference distributions, although some differences remain in the position and separation of the short-distance feature at low temperature. These results indicate that the quantum nuclear densities in CNEO recover important structural broadening effects that are absent from point-nucleus AIMD.

Because the H_2_–CH_3_ motif is no longer present, analysis of the dynamics requires a revised protocol. Using low-pass-filtered C–H bond-length descriptors together with a Gaussian mixture model, we developed a classification scheme that distinguishes near-equilibrium configurations from fluxional configurations based on the two-leg signature and identifies a transition-state-hovering pattern through the prolonged persistence of a single short C–H bond. Although hydrogen scrambling is expected even at low temperature for CH_5_^+^, CNEO-MD shows almost no scrambling events at 20 K, reflecting the limitations of the present classical dynamics treatment and finite trajectory length, especially when tunneling and large quantum delocalization are present. At 50 K, CNEO-MD predicts much more frequent hydrogen rearrangement, with substantial fluxional motion and extended residence in transition-state-hovering configurations. These results illustrate how the CNEO effective surface changes the apparent hydrogen-rearrangement dynamics relative to conventional point-nucleus descriptions.

For vibrational spectra, the more symmetric C_2v_ structure as the effective minimum leads to subtle but qualitative differences in mode assignments. CNEO harmonic analysis predicts systematic redshifts, particularly for C–H stretching modes and yields a low-frequency head-site proton-transfer-like mode below 500 cm^–1^. Temperature-dependent CNEO-MD spectra show progressive peak broadening and merging at elevated temperatures as anharmonicity, intermode coupling effects, and scrambling become more significant. At higher temperatures, differences from the experimental spectra become more pronounced. These differences likely reflect the limitations of applying classical dipole-autocorrelation analysis, which relies on approximate harmonic quantum–classical correspondence, to strongly fluxional and nonlocal dynamics on the CNEO effective surface.

Taken together, these results suggest that zero-point effects captured by CNEO can qualitatively influence the effective structural minimum and scrambling behavior of CH_5_^+^, with corresponding consequences for spectral interpretation. Further calculations, including extensions to deuterated isotopologues, could provide a natural next step and help assess whether the proposed structural picture, as well as the associated spectroscopic and dynamical signatures, persist under isotope substitution.

## Supplementary Material





## Notes

Optimized geometries of CH_5_^+^ obtained from CNEO–DFT calculations; harmonic vibrational frequencies of the global minimum and transition state; vibrational normal modes at the global minimum geometry; benchmark of exchange-correlation functionals and basis sets; Gaussian-mixture-model (GMM) fitting performed on unfiltered C–H bond lengths; structural evolution during one scrambling event; GMM predictions along a transition-state hovering trajectory (PDF)
Transition-state hovering animation (ZIP)
