# Supplementary material for: Assessing Constrained Nuclear–Electronic Orbital Theory for the Floppy CH 5 + Ion: Geometry-Dependent Zero-Point Effects on Structure, Hydrogen Rearrangement Dynamics, and Simulated IR Spectra
Source: J Phys Chem A. 2026 Jul 27;130(31):5978–92. doi: 10.1021/acs.jpca.6c01211 (PMC13455066; doi:10.1021/acs.jpca.6c01211)
Supplement: Supplementary file 1 [file jp6c01211_si_001.pdf]

Supporting Information: Assessing Constrained Nuclear–Electronic Orbital Theory for the Floppy CH_5^+ Ion: Geometry-Dependent Zero-Point Effects on Structure, Hydrogen Rearrangement Dynamics, and Simulated IR Spectra

Yiwen Wang, Zehua Chen, Yuzhe Zhang, Edwin L. Sibert III, Yang Yang*

Theoretical Chemistry Institute and Department of Chemistry, University of Wisconsin-Madison, 1101 University Avenue, Madison, Wisconsin 53706, United States

E-mail: yyang222@wisc.edu

S1. CNEO-DFT Optimized Geometries for Global Minimum and Transition State

Table S1. Interatomic distances, bond angles, and dihedral angles for the effective global-minimum (GM) with C_{2v} symmetry and transition-state (TS) structure with staggered-C_s symmetry of CH_5^+, optimized using CNEO-DFT (ωB97MV/def2-TZVPP). Hydrogen labeling follows Figure 2a in the main paper for the effective GM and Figure 2b in the main paper for the TS.

	Bond Length (Å)			Angle (°)	
	GM	TS		GM	TS
C-H1	1.160	1.135			
C-H2	1.186	1.172	ϕ(C-H1-H2-H3)	0	
C-H3	1.160	1.172	ϕ(H2-H4-H5-C)	0	
C-H4	1.113	1.135	ϕ(H1-C-H2-H4)	90	
C-H5	1.113	1.106			
H1-H2	1.258	1.505	∠H1-C-H2	65	81
H1-H3	2.100	2.074	∠H1-C-H3	130	128
H1-H4	1.768	1.666	∠H1-C-H4	102	94
H1-H5	1.768	1.900	∠H1-C-H5	102	116
H2-H3	1.258	1.223	∠H2-C-H3	65	63
H2-H4	1.988	2.074	∠H2-C-H4	120	128
H2-H5	1.988	1.891	∠H2-C-H5	120	112
H3-H4	1.768	1.505	∠H3-C-H4	102	81
H3-H5	1.768	1.891	∠H3-C-H5	102	112
H4-H5	1.936	1.900	∠H4-C-H5	121	116

S2. Harmonic Analysis Results

Table S2. Harmonic vibrational frequencies (in cm^{-1}) from CNEO-DFT (ωB97MV/def2-TZVPP) for the effective global-minimum (GM) with C_{2v} symmetry and transition-state (TS) structure with staggered-C_s symmetry of CH_5^+. The TS structure has one imaginary frequency, consistent with a first-order saddle point.

GM	151	330	1056	1230	1340	1368	1381	2503	2609	2769	2966	3065
TS	181i	356	928	1204	1345	1345	1385	2543	2611	2849	2862	3088

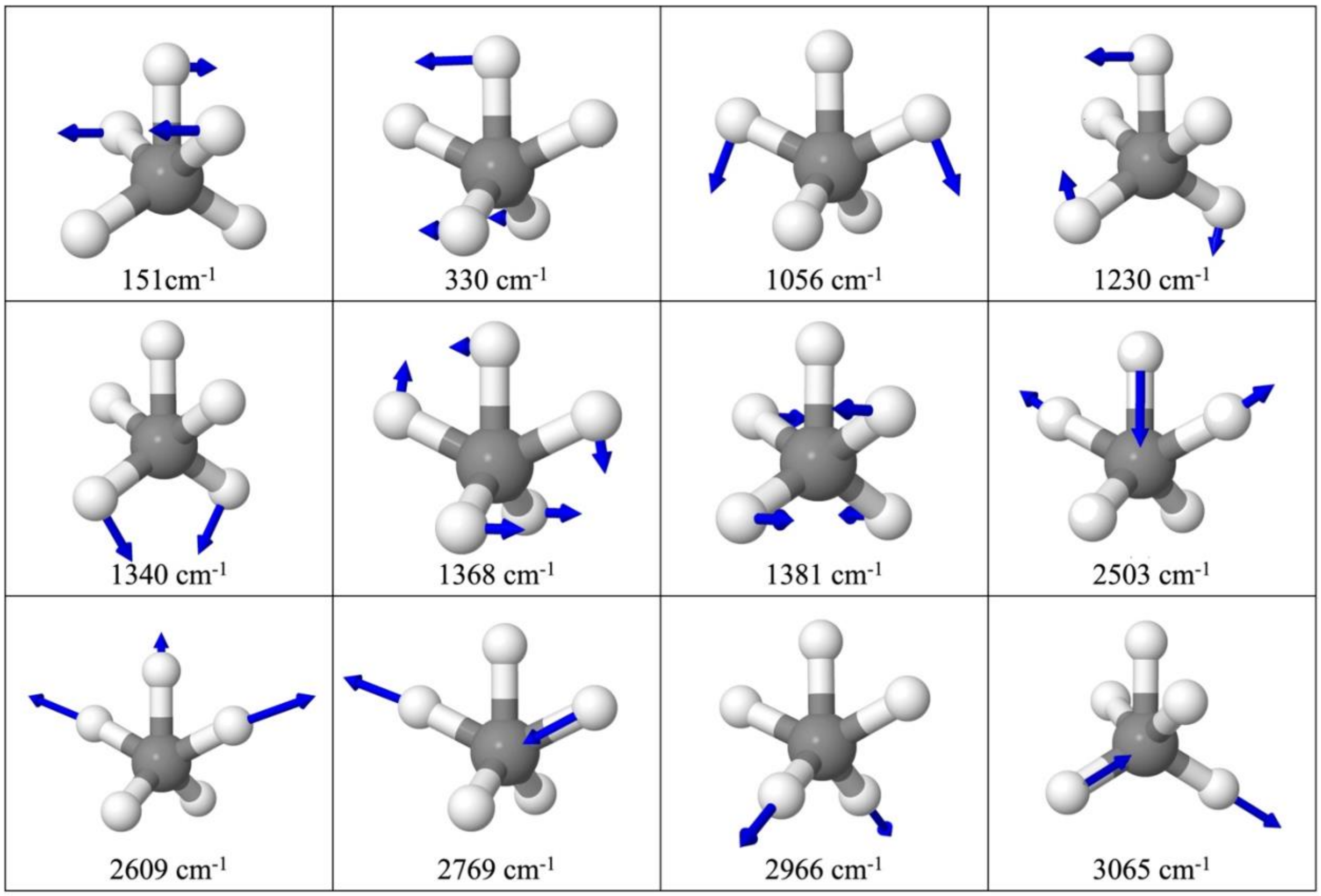

Figure S1. Visualization of the vibrational normal modes of CH_5^+ from CNEO-DFT harmonic analysis. The harmonic vibrational frequencies are computed at ωB97MV/def2-TZVPP level.

S3. Benchmarking of Functionals and Basis Sets

To minimize errors associated with the choice of exchange–correlation functional and electronic basis set, conventional DFT harmonic vibrational frequencies of CH_5^+ were benchmarked against CCSD(T) reference data. CCSD(T) reference values are generated with both def2-TZVPP and aug-cc-pVTZ basis sets. The frequencies and corresponding root-mean-square deviations (RMSDs) are summarized in Table S3. The RMSDs obtained with the two CCSD(T) reference sets are very similar.

Among the methods tested, ωB97M-V/def2-TZVPP shows good agreement with the CCSD(T) results. Additional benchmarks using ωB97M-V calculations with larger basis sets indicate that basis-set enlargement yields only modest improvements in the harmonic frequencies. In particular, the RMSD changes by less than a few cm^{-1} when moving from def2-TZVPP to aug-cc-pVQZ-quality basis sets. Therefore, ωB97M-V/def2-TZVPP provides a favorable balance between accuracy and computational cost and is used for all calculations reported in the main text.

Table S3. Harmonic vibrational frequencies (in cm^{-1}) from conventional DFT using different functionals and basis sets, benchmarked against CCSD(T) reference data.

	CCSD(T) def2-TZVPP	CCSD(T) aug-cc-pVTZ	PEB0 aug-cc-pVDZ	PBE0 def2-TZVPP	ωB97M-V aug-cc-pVDZ	ωB97M-V def2-TZVPP	ωB97M-V aug-cc-pVTZ	ωB97M-V aug-cc-pVQZ
	181	253	266	238	262	218	218	208
	815	815	441	576	456	628	635	652
	1290	1293	1174	1205	1174	1213	1214	1217
	1303	1304	1278	1289	1268	1281	1281	1281
	1472	1475	1408	1431	1401	1430	1431	1433
	1488	1505	1436	1468	1427	1466	1468	1468
	1589	1595	1446	1493	1438	1499	1502	1506
	2443	2443	2608	2576	2571	2524	2515	2513
	2712	2714	2704	2677	2694	2667	2662	2663
	3013	3012	2967	2977	2961	2985	2982	2984
	3136	3132	3133	3126	3129	3127	3123	3124
	3232	3224	3245	3227	3242	3228	3224	3223
RMSD[1]	0	22	135	91	130	72	70	65
RMSD[2]	22	0	134	91	129	74	71	67

[1] RMSD with reference value from CCSD(T)/def2-TZVPP calculations
[2] RMSD with reference value from CCSD(T)/aug-cc-pVTZ calculations

S4. Gaussian-Mixture-Model Fitting of Unfiltered C-H Bond Lengths

In principle, the unfiltered C–H bond lengths can also be used to build the GMM. The C–H bond-length distribution from the 20 K simulations is shown in Figure S2, where the shaded region represents the raw data collected from the trajectories. The GMM fit using three Gaussian components is shown as the three colored curves, and the total fitted distribution is shown as the black curve. The fitted Gaussian centers are 1.115, 1.156, and 1.180 Å, which closely match the characteristic C–H bond lengths of the C_{2v} global-minimum structure (1.113, 1.160, and 1.186 Å).

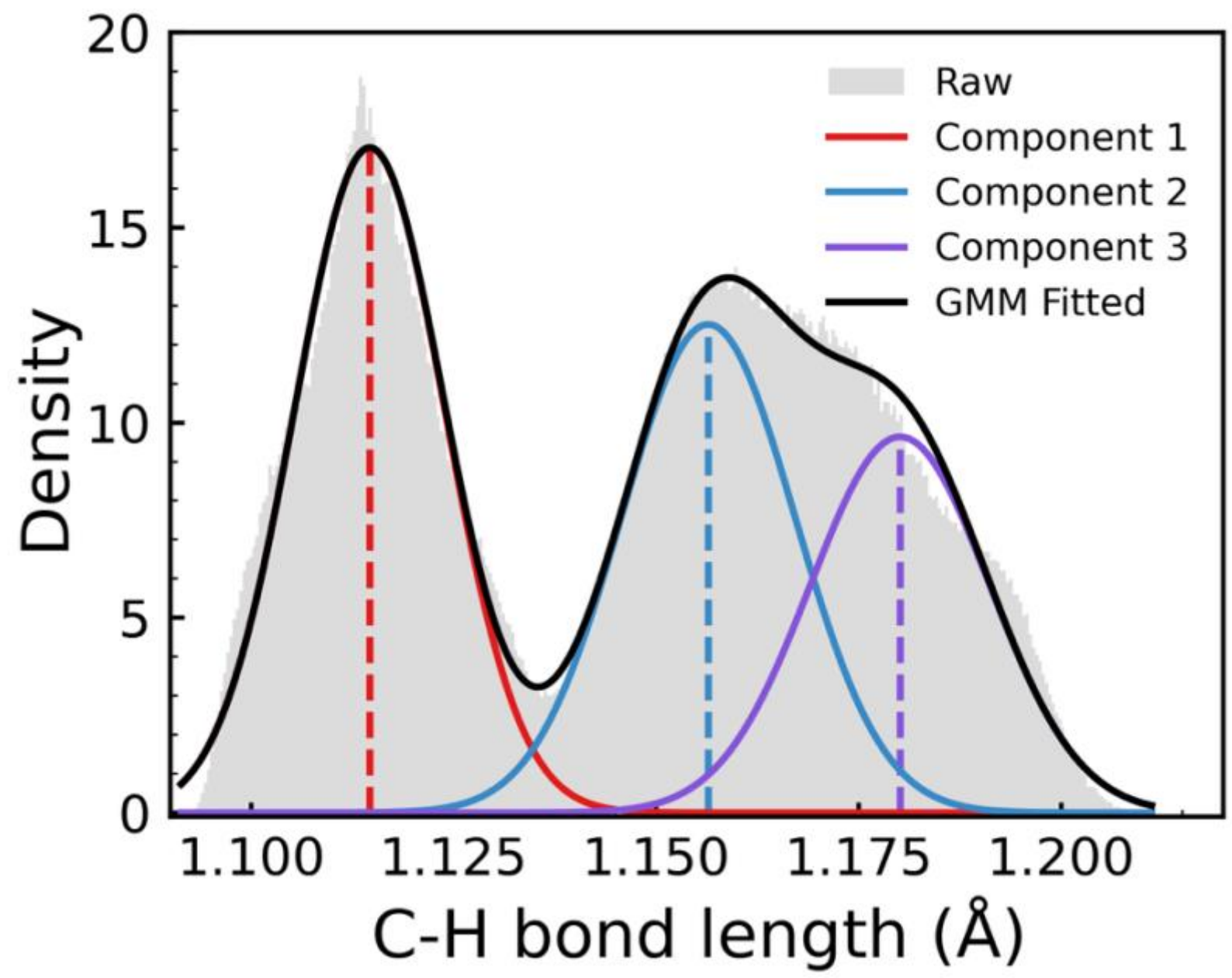

Figure S2. Unfiltered C–H bond-length distribution and GMM fit. The shaded region shows the C–H bond-length data collected from 50 trajectories at 20 K (4000 configurations per trajectory), yielding a total of 20,000 bond lengths. The three Gaussian components are shown as colored curves, and their sum is shown as the solid black curve, corresponding to the overall fitted distribution.

S5. Structural Evolution During One Scrambling Event

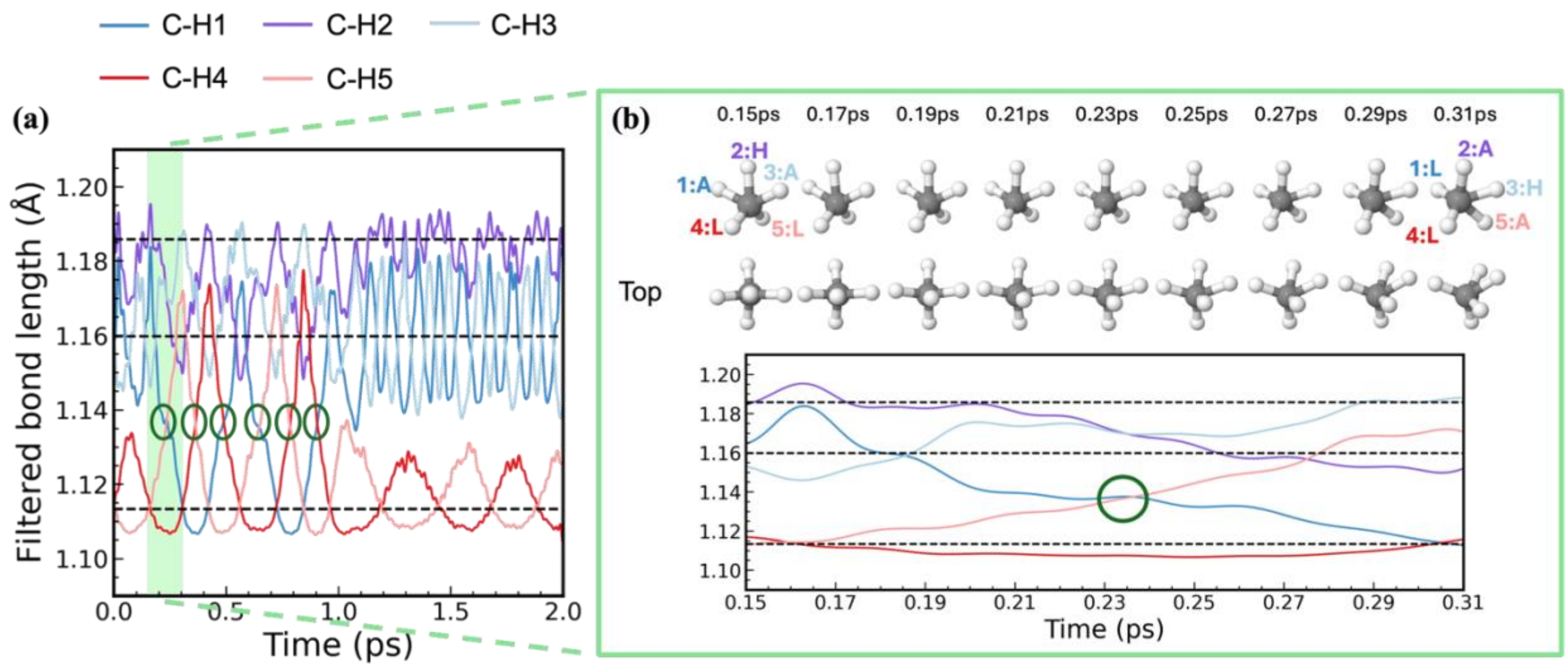

Figure S3. (a) Five filtered C–H bond lengths along a representative 50 K trajectory. The solid green circles highlight region-crossing events. Hydrogen indices follow the labeling in Figure 2 of the main paper. **(b)** Enlarged view of the shaded interval in panel (a) (0.15–0.31 ps). The corresponding structural evolution (front and top views) is shown above at 0.02 ps intervals. Hydrogen indices and site identities for the initial and final structures are labeled, where H denotes the head site, A the arm sites, and L the leg sites. This evolution illustrates that a hydrogen-scrambling event occurs during the region-crossing pattern. In both panels, the black dashed lines indicate the three characteristic C–H bond lengths of the C_{2v} global-minimum structure.

S6. GMM Predictions During Transition-State Hovering

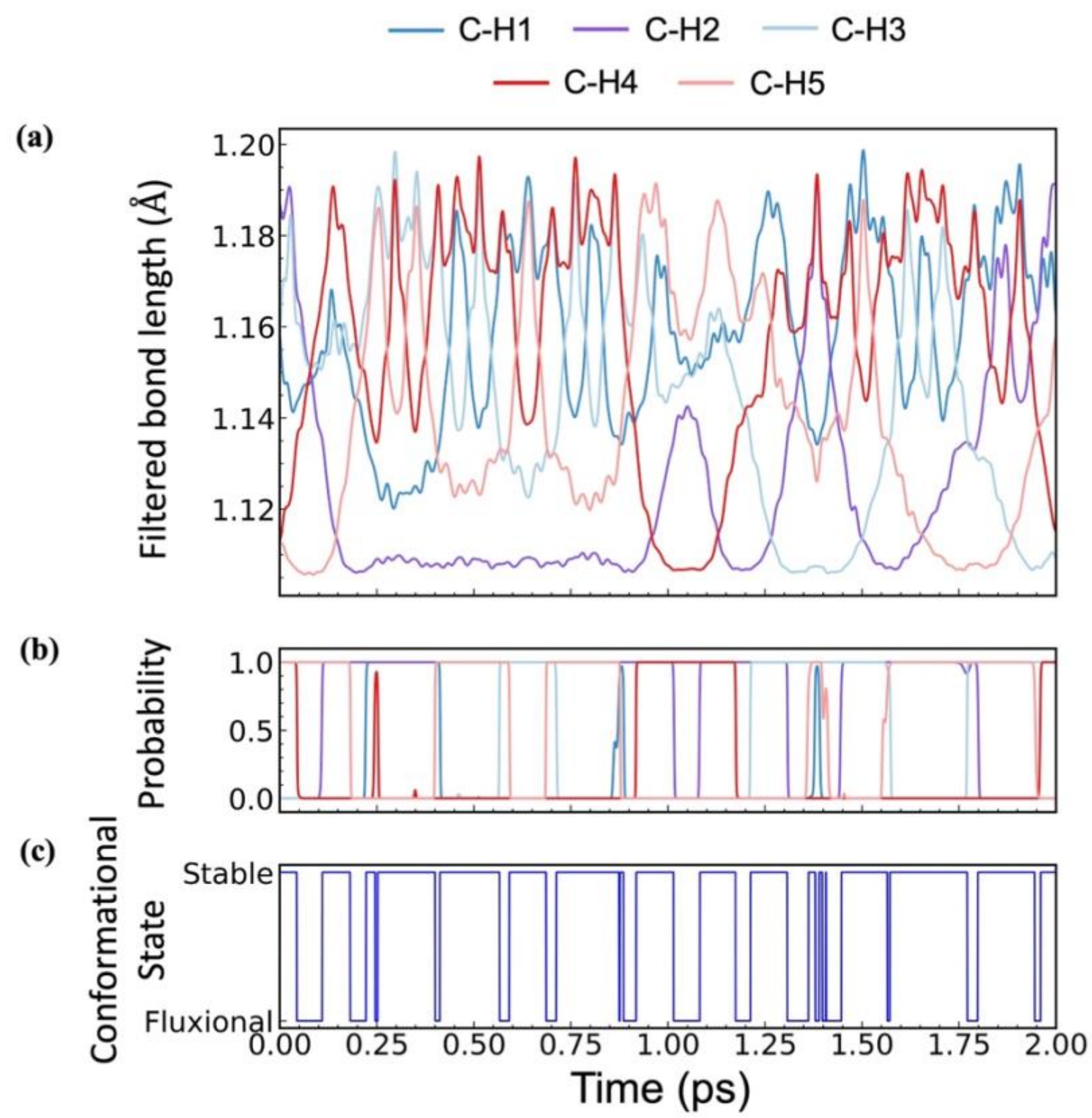

Figure S4. GMM-based scrambling analysis for a representative 50 K trajectory exhibiting transition-state hovering. **(a)** Filtered C–H bond lengths for all five hydrogens; the 0.2–0.9 ps segment corresponds to the transition-state-hovering dynamical pattern described in the main paper. **(b)** GMM-predicted probabilities that each bond corresponds to one of the two leg sites. **(c)** Conformational assignments obtained by counting the number of bonds whose leg-site probability exceeds the chosen cutoff. Hydrogen indices follow the labeling scheme in Figure 2 of the main paper.

Figure S4 shows the GMM-based scrambling analysis for the representative trajectory that exhibits the transition-state-hovering dynamical pattern. Panel (c) reports the conformational assignments obtained using only instantaneous structural features. In the time window later identified as transition-state hovering (0.2–0.9 ps), most frames are labeled as stable, with occasional transitions assigned as fluxional.

However, direct visualization of the trajectory indicates that these assignments are not reliable. During this period, the structure rarely exhibits the three-hydrogen coplanar arrangement characteristic of the C_{2v} global-minimum configuration. Instead, the geometries closely resemble the transition-state structure, with the shortest C–H bond lying in the mirror plane while the remaining four hydrogens interchange their roles with large bond-length fluctuations. This misclassification motivates the identification of the transition-state-hovering dynamical feature described in the main text, which is based on the prolonged "one-bond/four-bond" signature.
